# Dimensions of inattention: Cognitive, behavioral, and affective consequences

**DOI:** 10.3389/fpsyg.2023.1075953

**Published:** 2023-02-28

**Authors:** Jennifer M. Yip, Natalie M. Jodoin, Todd C. Handy

**Affiliations:** Department of Psychology, University of British Columbia, Vancouver, BC, Canada

**Keywords:** inattention, mind-wandering, daydreaming, repetitive negative thoughts, perseverative cognition, depression, anxiety, mood

## Abstract

Inattention to one’s on-going task leads to well-documented cognitive, behavioral, and physiological consequences. At the same time, the reliable association between mind-wandering and negative mood has suggested that there are affective consequences to task inattention as well. We examined this potential relationship between inattention and mood in the following study. Six hundred and fifty-five participants completed self-report questionnaires related to inattentive thinking (i.e., attentional lapses, daydreaming, mindfulness, rumination, reflection, worry, postevent processing, inattentiveness, and counterfactual thinking), a questionnaire about depressive symptoms, and a questionnaire about anxiety symptoms. First, an exploratory factor analysis was conducted to identify potential underlying constructs of types of inattentive thinking. Using ordinary least squares extraction and Oblimin rotation, a three-factor model demonstrated suitable fit, broadly representing mind-wandering/inattentive consequences, repetitive negative thinking, and reflective/introspective thinking. Second, after eliminating measures that did not strongly load on any factor, structural equation modeling was conducted and found that the relationship between mind-wandering and depression was *partially explained* by repetitive negative thinking, whereas the relationship between mind-wandering and anxiety was *fully explained* by repetitive negative thinking. The present findings suggest that understanding how inattentive thoughts are interrelated not only influences mood and affect but also reveals important considerations of intentionality, executive functioning, and qualitative styles of these thoughts.

## Introduction

Having our thoughts drift away from the task at hand is a common and relatable experience for all of us. From driving a familiar route home to washing the dinner dishes, our minds readily take us out of our immediate surroundings and into our internal dwellings. Sometimes our minds wander off to relatively neutral topics such as what we want to eat for our next meal, but other times we might be in a negative mood and what we think about follows suit, often in a recurring manner. For example, after an argument with a loved one, we might find ourselves repeating the conversation in our heads, perhaps about alternative scenarios or possible consequences of the talk, or even blaming the other person or ourselves. These are all co-occurrences or results of inattention; however, thought quality and content can differ greatly depending on context–– it can be an experience we inadvertently slip into, one that we embrace and enjoy, or one that we make every effort to push away.

In the past few decades, science has taken a keen interest in exploring the varied, multi-dimensional nature of inattention, and its study has been equally diverse. For example, while cognitive psychology has primarily investigated inattention from the perspective of mind-wandering (MW; e.g., Giambra, 1989; Schooler et al., 2011; Levinson et al., 2012), daydreaming (e.g., Lindquist and McLean, 2011; Stawarczyk et al., 2012; Poerio and Smallwood, 2016), and attentional lapses (e.g., Smallwood et al., 2004, 2009; McVay and Kane, 2009) as the defining constructs, clinical psychology has primarily focused on inattention from the perspective of rumination, post-event processing, and worry (e.g., Zetsche et al., 2012; Whitmer and Gotlib, 2013). However, although this work has proceeded somewhat independently between the two fields of study, might research in the two areas be converging on a common underlying cognitive dynamic?

In particular, MW as a cognitive phenomenon has been associated with negative cognitive, behavioral, autonomic (Ottaviani and Couyoumdjian, 2013; Ottaviani et al., 2015) and moods (Smallwood et al., 2009; Killingsworth and Gilbert, 2010), not unlike rumination and worry as a clinical phenomenon a point of convergence that mirrors clinical phenomena of rumination (see Watkins and Roberts, 2020 for a review), post-event processing (Kashdan and Roberts, 2007), and worry (see Brosschot et al., 2006 for a review). This in turn raises a critical question: Could repetitive negative thoughts (RNTs) as studied in the clinical domain explain or mediate the relationship between MW and negative moods as observed in the cognitive domain? Here we report the results of a questionnaire-based study that suggests the answer is *yes*.

RNTs are typically recurrent, excessive, and somewhat circular, and are prevalent in various psychopathologies. While worry is a common feature associated with general anxious affect (Borkovec et al., 1983), rumination is associated with depression (Nolen-Hoeksema and Morrow, 1991), and post-event processing with social anxiety disorder (Rachman et al., 2000). Nonetheless, these RNTs have been found to highly correlate with each other and researchers have disputed the utility of considering them as separate constructs (e.g., Watkins, 2008; McEvoy et al., 2010; Wahl et al., 2019). In contrast to MW, RNTs and their subtypes have been well-studied, and RNTs’ links with psychopathology and well-being have been well-established (for reviews, see Ehring and Watkins, 2008; Clancy et al., 2016; Zetsche et al., 2018).

Against this background, several studies to date have linked MW and negative moods. Using experience sampling methods to randomly probe for MW states either in the lab during an experiment or as participants go about their everyday lives has suggested that MW is associated with negative or depressive moods (see, e.g., Killingsworth and Gilbert, 2010; Smallwood and O'Connor, 2011; Franklin et al., 2013; Poerio et al., 2013; Ruby et al., 2013; Jonkman et al., 2017; Yamaoka and Yukawa, 2020). Similarly, chronic stress has been linked with more MW, and in turn, linked with lower positive mood and higher negative mood (Crosswell et al., 2020). But beyond these more direct links, the potential for RNTs to mediate the relationship between MW and negative moods is consistent with three more specific aspects of MW that may be fundamental and underlie other manifestations of inattention.

The first concerns the intentionality of inattention, or whether one is deliberately or spontaneously inattentive. Spontaneous periods of MW appear related to symptoms of depression, anxiety, and stress, whereas deliberate MW very weakly correlated with these same symptoms (Seli et al., 2019). In line with this MW intentionality division, problematic gamblers tended to deliberately mind-wander to cope with negative affect (likely boredom) induced by a vigilance task but showed no such pattern for a more engaging gambling task (Kruger et al., 2020). To wit, self-rumination, a form of RNT, appeared to predict spontaneous MW, and self-reflection appeared to predict deliberate MW (Vannucci and Chiorri, 2018), and spontaneous MW and self-rumination appear to mediate the relationship between high levels of self-consciousness and depressive symptoms (Scalabrini et al., 2022). Relatedly, a review examining MW, negative mood, and depression suggested that interrelationships and mixed findings may be explained by the state of mind even during MW. Specifically, Konjedi and Maleeh (2017) proposed that MW without meta-awareness (i.e., thinking on autopilot) or with excessive vigilance about thought content may lead to more negative mood, whereas MW *with* meta-awareness (e.g., during meditation), in direct contrast, actually does *not* lead to negative mood. Still, both “zone-outs” (MW without awareness) and “tune-outs” (MW with awareness) have been positively correlated with depressive symptoms, suggesting that having greater depressive symptoms in general is linked with MW more often irrespective of the presence or absence of meta-awareness. Similarly, those who have greater depressive symptoms appear to mind-wander with awareness more often (Nayda and Takarangi, 2021), which may align with the proposition that these depressive MW thoughts may have an excessively vigilant quality about the content of what one is thinking about.

Second, intentionality and goal-driven behaviors, including selecting where we direct our internal attention, requires our executive control. Both research from a cognitive, MW perspective and research from a clinical, RNTs perspective both point to executive functioning mechanisms as contributors to inattention. For example, MW may enlist executive function resources that have a limited capacity, trading off between performing a task or engaging in task-unrelated thoughts (Smallwood and Schooler, 2006). Likewise, MW may represent an executive function “failure,” momentarily, in that it is a diminished ability to deter task-unrelated interferences (McVay and Kane, 2010). In turn, repetitive thought researchers have proposed that repetitive thought occurs until some current concern or personal goal is either “resolved or abandoned” (Klinger, 1971, 2012), and that executive function is specifically involved to bring abstract goals to a concrete level, which is necessary to meet the challenges of novel or difficult tasks (Watkins, 2008). Clinical RNT researchers have recognized executive functioning deficits as one mechanism that contributes to impaired problem-solving, getting stuck in habits, and as suggested above, an inability to shift out of abstract thinking into concrete problem-solving during rumination (Watkins and Roberts, 2020). Thus, executive control may manage the kinds of thoughts that enter the forefront of our minds and whether we can translate them into problem-solving goals (McVay and Kane, 2010). Yet, reviews have suggested that RNT stems specifically from difficulty in ignoring information in working memory that is no longer relevant for goal-directed behavior (Whitmer and Gotlib, 2013; Zetsche et al., 2018), which may be aligned to an extent with the executive failure hypothesis (McVay and Kane, 2010).

Finally, there is the qualitative content of inattentive thoughts themselves. Given that we mind-wander upwards of 50% of our lives (Killingsworth and Gilbert, 2010), unsurprisingly, the content of task-unrelated thinking is most often autobiographical and related to planning for our future in some manner (Baird et al., 2011). Indeed, more research has explored the temporal orientation in the content of MW thoughts, which may be one additional factor in the relationship between MW and mood. Mind-wandering about past events appears to occur more frequently when it is preceded by induced negative mood (Smallwood and O'Connor, 2011) or when negative mood is already present (Poerio et al., 2013); however, even after controlling for sad mood preceding MW, MW also seems to predict later mood (Ruby et al., 2013), though this effect may not last longer than 15 min (Poerio et al., 2013). Moreover, the more past-oriented MW, the higher depressive symptoms tend to be (Smallwood and O'Connor, 2011). One study had a somewhat unexpected finding that state MW temporal orientation during a task was unrelated to trait rumination (Shrimpton et al., 2017), despite that rumination comprises recurrent, depressive thoughts related to unchangeable situations in the past. Yet having a past temporal orientation was still associated with poorer task performance (Shrimpton et al., 2017). Mind-wandering about the future has also been found as common (Poerio et al., 2013), with at least one study linking future-oriented MW with positive moods or thinking styles (Shrimpton et al., 2017). In this respect, we may consider synthesizing research on worry that is anxiety-driven and often comes with much planning about the future or getting stuck on “what if” thoughts.

### Objectives

This study has the overarching aim of examining whether affective consequences can account for cognitive and behavioral consequences of inattention. To do so, we examine common dimensions or characteristics of inattention, particularly in the MW domain and recurrent thoughts. Manifestations of inattention included in this study are construed as attentional lapses, daydreaming, mindfulness, rumination, reflection, worry, postevent processing, ADHD inattentiveness, and counterfactual thinking. Measures of attentional lapses, daydreaming, and mindfulness (reversed scored) have commonly been used to assess MW on a trait level, though each scale likens MW slightly differently: *daydreaming* is thinking that is shifted away from a current task or an external situation, typically thought of as more intentional than mind wandering (Singer and Antrobus, 1963); *mindfulness* is the state of being attentive and aware of the present moment (Brown and Ryan, 2003); *attentional lapses*, or absent-mindedness, are demonstrated by minor negative repercussions of being momentarily off-task (e.g., walking into a room and forgetting what one intended to do; Carriere et al., 2008).

Measures of recurrent thoughts are described as follows: *Rumination* is repetitively thinking about an experience in a negative light after the experience occurred (Nolen-Hoeksema and Morrow, 1991) or about the level of neurotic self-consciousness; *reflection* measures the level of intellectual self-consciousness to a given situation (Trapnell and Campbell, 1999); *worry* is repetitively thinking about future life events with marked anxiety about uncertainty of outcomes (Roemer and Borkovec, 1993); *post-event processing* is repeatedly thinking about a perceived inadequacy in a previous social situation (McEvoy and Kingsep, 2006); *inattentiveness* is difficulty concentrating and *hyperactivity-impulsivity* is characterized by restlessness, fidgeting, and behavioral inhibition, which may be reflective of poor executive functioning or mental control; *counterfactual thinking* is conceiving of alternatives to how reality unfolded in the recent past, such as “if only” thoughts (Rye et al., 2008).

The first main objective is to explore how these manifestations of inattention converge and overlap with each other; i.e., similarities and differences that may be demonstrated in a certain number of latent constructs. The second main objective is to determine how much of the relationship between MW and low or anxious mood may be explained by recurrent thoughts.

## Methods

### Participants

Six hundred and fifty-five participants were recruited using Amazon’s Mechanical Turk (MTurk), an Internet crowd-sourcing marketplace where researchers can post studies for participants to complete. Data collected *via* MTurk is comparable to data collected by traditional means with regards to participant characteristics, attrition, effect sizes, validity, and reliability (e.g., Casler et al., 2013; Schleider and Weisz, 2015; Ramsey et al., 2016). Moreover, MTurk has the advantages of having stable access to a diverse group of individuals (e.g., Buhrmester et al., 2011) as well as being more cost-effective than laboratory studies (Mason and Suri, 2012; Schleider and Weisz, 2015). Previous MW studies have found the medium to be well-suited to collecting data (e.g., Carriere et al., 2013).

Several steps were taken to maximize data validity based on recommendations in the literature (e.g., Aust et al., 2013; Rouse, 2015; Thomas and Clifford, 2017). Ex ante exclusion involved initial screener questions consisting of the two eligibility criteria, *via* a drop-down list to select age and primary language, which needed to be above 18 and English in order to proceed with the study. The study was described as a “psychology survey about your thought patterns, self-reflection, and attention” and described it as a “UBC-based study: make ratings about your thought patterns, a 30–40-min survey,” which addresses one of MTurkers’ complaints of unclear task description such as not providing an estimated time commitment. The participant requirement standard was set to the highest available on MTurk: only those with a system qualification of >99% approval rate on more than 10,000 “Human Intelligence Tasks” can access the study. Participants who met those criteria were asked to complete a set of questionnaires hosted on the online platform, Qualtrics. The time estimate of 30–40 min was based on timing three fluent English-speaking undergraduate students; therefore, for a broader audience, 1 h was allotted to complete all questionnaires. Each participant was paid $1.75 based on studies at the time of data collection that evaluated the balance of compensation, data collection rate, motivation, and data quality (Bohannon, 2011; Buhrmester et al., 2011; Litman et al., 2015). Ex post exclusion involved eliminating from analyses participants who: (1) did not score 3 out of 3 on attention check items that were embedded in three questionnaires (e.g., for this question, please select the response option “frequently”), (2) had more than 10 measure items missing, and (3) were outliers in response times, i.e., responding too quickly or too slowly. Although other screeners could be included, such as comprehension and/or open-ended questions, we opted for keeping with the above checks in order to minimize de-incentivizing participants’ efforts and time. Notably, our data was collected in 2016, prior to the observed spike in fraudulent responses by use of virtual private servers (see Dennis et al., 2020) in the summer of 2018 (Chmielewski and Kucker, 2020; Kennedy et al., 2020). Six hundred and twelve participants remained and were included in the final data analyses of this study.

### Measures

The following measures are solely based on participants’ self-report; therefore, we are assessing participants’ own reflections on all their thought tendencies and correlates, rather than a direct measurement of inattention *per se*.

#### Inattention questionnaires

The Attention-Related Cognitive Errors Scale (ARCES) is a 12-item self-report measure of how often one makes mistakes as a result of lapses in attention to their daily activities (e.g., *I have lost track of a conversation because I zoned out when someone else was talking*; Carriere et al., 2008). The ARCES uses a five-point Likert scale ranging from 1 (*never*) to 5 (*very often*). Higher scores are indicative of more attentional lapses. Psychometric properties of the ARCES reveal strong internal consistency (Cronbach’s alpha = 0.88; Carriere et al., 2008). Furthermore, this scale demonstrates sufficient convergent and construct validity (Cheyne et al., 2006).

The Daydreaming Frequency Scale (DFS) is a 12-item self-report measure of how often one daydreams during their daily activities and in their general life (e.g., *When I am not paying close attention to some job, book, or TV, I tend to be daydreaming*; Singer and Antrobus, 1963, 1970, 1972). The DFS uses a five-option multiple choice format ranging from A (least amount of daydreaming) to E (most amount of daydreaming). Higher scores are indicative of more daydreaming. The DFS demonstrates relatively strong reliability (Cronbach’s alpha = 0.91), and test–retest reliability (Cronbach’s alpha = 0.76; Giambra, 1993). This scale has also shown relatively robust validity (Stawarczyk et al., 2012).

The Mindful Attention and Awareness Scale (MAAS) is a 15-item self-report measure of how often one is conscious of their present surroundings and experiences in everyday life (e.g., *I could be experiencing some emotion and not be conscious of it until some time later*; Brown and Ryan, 2003). The MAAS uses a six-point Likert scale ranging from 1 (*almost always*) to 6 (*almost never*). Higher scores on the MAAS indicates more mindfulness. The MAAS shows strong internal consistency (Cronbach’s alpha = 0.92) and strong validity reported by the initial study (Brown and Ryan, 2003).

The Current Symptoms Scale – Self-Report Form (CSS) is an 18-item self-report measure of ADHD based on DSM-IV symptoms of inattentiveness and hyperactivity/impulsivity (e.g., *I have difficulty sustaining my attention in tasks or fun activities*). The CSS uses a four-point Likert scale ranging from 0 (*never or rarely*) to 3 (*very often*). Higher scores are indicative of greater ADHD symptoms. The CSS can be separated into subscales of inattentiveness (CSS-IA) and hyperactivity/impulsivity (CSS-HI). Internal consistency Cronbach’s alpha ranges 0.75–0.91 (Taylor et al., 2011; Gomez et al., 2021).

The Counterfactual Thinking for Negative Events Scale (CTNES) is a 16-item self-report measure of how often one thinks about alternate outcomes of a recent event that differs from what had occurred in reality (e.g., *I think about how much worse things could have been*; Rye et al., 2008). This CTNES uses a five-point Likert scale ranging from 1 (*never*) to 5 (*very often*). Higher scores reflect more counterfactual thoughts. The CTNES demonstrates relatively strong test–retest and inter-rater reliability as well as strong validity (Rye et al., 2008).

The Penn State Worry Questionnaire (PSWQ) is a 16-item self-report measure of one’s tendency to worry, including how often one worries and how uncontrollable worries are perceived to be (e.g.*, I know I should not worry about things, but I just cannot help it*; Meyer et al., 1990). The PSWQ uses a five-point Likert scale ranging from 1 (*not at all typical of me*) to 5 (*very typical of me*). Higher scores are indicative of greater tendency to worry. The PSWQ has been found to have very strong internal consistency, and satisfactory test–retest reliability (Meyer et al., 1990). Additionally, the PSWQ demonstrates strong validity, in both the original investigation (Meyer et al., 1990) and across a variety of studies (see Molina and Borkovec, 1994).

The Post-Event Processing Questionnaire Revised (PEPQ-R) is a 14-item self-report measure of negative thoughts and emotions related to dwelling on a recent past social situation or interaction that evoked anxiety, related to one’s perceived performance or demeanor (e.g., *Did the thoughts about the event ever interfere with your concentration?*; McEvoy and Kingsep, 2006). The PEPQ-R uses a visual analogue scale ranging from 0 (*not at all*) to 100 (*totally agree*). Higher scores are indicative of more post-event processing. The PEPQ-R has demonstrated good internal reliability (Cronbach’s alpha = 0.87) and has been found to appropriately measure types of anxiety such as social phobias (McEvoy and Kingsep, 2006).

The Ruminative Responses Scale (RRS) of the Response Style Questionnaire is a 20-item self-report measure of negative recurring thinking tendencies about oneself and one’s low mood (e.g., *When you feel down, sad, or depressed, you think about how alone you feel*; Nolen-Hoeksema and Morrow, 1991). The RRS uses a four-point Likert scale ranging from 1 (*almost never*) to 4 (*almost always*). Higher scores on the RRS are indicative of more rumination. The RRS can be separated into subscales of brooding (RRS-Brood), reflection (RRS-Refl), and depression. This scale has demonstrated strong internal consistency (Cronbach’s alpha = 0.89), and strong validity (Nolen-Hoeksema and Morrow, 1991). Treynor et al. (2003) demonstrated that the depression subscale has substantial overlap with depressive symptom scales such as the Beck Depression Inventory (Beck et al., 1996) and thus advocate for using the 10 items constructed from the brooding (RRS-Brood) and reflection (RRS-Refl) subscales only, and this RRS-10 version was used for analyses in the current study.

The Rumination and Reflection Questionnaire (RRQ) is a 24-item self-report measure of negative recurring thinking tendencies about oneself and one’s low mood and introspective tendencies (e.g., *I always seem to be rehashing in my mind recent things I’ve said or done;* Trapnell and Campbell, 1999). The RRQ uses a five-point Likert scale ranging from 1 (*strongly disagree*) to 5 (*strongly agree*). Higher scores are indicative of more rumination or reflection. The RRQ can be separated into subscales of rumination (RRQ-Rum) and reflection (RRQ-Refl). The RRQ demonstrates acceptable validity and strong reliability (Cronbach’s *α* = 0.90–0.91; Trapnell and Campbell, 1999).

#### Symptoms questionnaires

Center for Epidemiologic Studies Depression Scale (CES-D) is a 20-item self-report measure of depressive symptomatology (e.g., *I was bothered by things that usually do not bother me*; Radloff, 1977). The CES-D uses a four-point Likert scale ranging from 0 (*rarely or none of the time*) to 3 (*most or all of the time*). Higher scores are indicative of more severe depressive symptoms. The CES-D has good internal consistency (α ≥ 0.80; Radloff, 1977; 0.90–0.93; Verdier-Taillefer et al., 2001), moderate test–retest correlations (*r* ≥ 0.40) ranging between 2 weeks and 12 months between tests, and construct and discriminant validity has been established across numerous other measures, including clinical ratings of depression and self-report questionnaires (Radloff, 1977).

The State–Trait Anxiety Inventory (STAI) is a 40-item self-report measure of anxiety at a particular moment in time (state) and of anxiety that one generally feels (e.g., *I feel that difficulties are piling up so that I cannot overcome them*; Spielberger, 1983). The STAI uses a four-point Likert scale ranging from 1 (*almost never*) to 4 (*almost always*). Higher scores are indicative of more severe anxiety symptoms. The STAI can be separated into subscales of state anxiety and trait anxiety. The STAI has good internal consistency (*α* = 0.83–0.92), high test–retest reliability for the trait subscale (*r* = 0.73–0.86), low reliability as expected for the state subscale (*r* = 0.16–0.54; due to situational factors influencing state), and good concurrent validity (*r* = 0.41–0.84; Spielberger, 1983).

## Results

### Objective 1

To identify which unique aspects of inattention converge with each other, exploratory factor analysis (EFA) was used. EFA re-arranges variables into a small set of clusters based on shared variance and allowing underlying constructs to be isolated. Shared variances infer common underlying factors that influence two or more surface constructs, whereas nonshared variances infer specific factors are unique to a certain surface construct (Yong and Pearce, 2013). An EFA parceled by measures was conducted to examine which manifestations of inattention included in our study (i.e., rumination, reflection, worry, postevent processing, inattentiveness, counterfactual thinking, daydreaming, mindfulness, and attentional lapses) cluster together using mean scale scores.

#### Finding 1

The *psych* package in R was used for the following data analysis.

Prior to the main exploratory factor analysis, the data matrix was evaluated. Evaluating individual variables *via* the Shapiro–Wilk normality test (all *p* < 0.001) and graphical representations (i.e., histograms, stem and leaf, box plots) concurrently suggest the data is not normally distributed. Additionally, a comparison between Pearson’s r and Spearman’s rho was conducted and showed that while many values were similar, there were several more values in which Spearman’s was significantly higher than Pearson’s correlation, supporting the use of ordinary least squares estimator in the subsequent exploratory factor analysis to appropriately handle non-normal data with relatively more accurate parameter estimates (Lee et al., 2012; Coughlin, 2013). See Table 1 for Spearman’s rho bivariate correlations.

**Table 1:** Bivariate correlations (Spearman’s rho) of inattention variables and symptom measures.

	ARCES	DFS	MAAS	CSS-IA	CSS-HI	CTNES	PEPQ-R	PSWQ	RRQ-Rum	RRQ-Refl	RRS-Brood	RRS-Refl	CES-D	STAI	*Mean*	*SD*
ARCES	–														2.43	0.78
DFS	0.493**	–													2.58	0.95
MAAS	0.612**	0.410**	–												2.70	0.97
CSS-IA	0.662**	0.471**	0.618**	–											1.56	0.57
CSS-HI	0.591**	0.426**	0.552**	0.772**	–										1.60	0.52
CTNES	0.445**	0.372**	0.386**	0.409**	0.419**	–									2.72	0.698
PEPQ-R	0.448**	0.446**	0.403**	0.514**	0.490**	0.464**	–								38.35	20.74
PSWQ	0.440**	0.420**	0.417**	0.474**	0.489**	0.369**	0.526**	–							2.97	1.03
RRQ-Rum	0.470**	0.508**	0.455**	0.468**	0.460**	0.487**	0.613**	0.755**	–						3.11	0.97
RRQ-Refl	−0.029	0.236**	−0.036	−0.073	−0.059	0.136**	0.067	−0.015	0.116	–					3.24	0.83
RRS-Brood	0.464**	0.391**	0.428**	0.560**	0.539**	0.590**	0.582**	0.586**	0.637**	0.030	–				2.03	0.78
RRS-Refl	0.374**	0.381**	0.323**	0.435**	0.425**	0.464**	0.464**	0.314**	0.398**	0.317**	0.612**	–			1.95	0.68
CES-D	0.555**	0.444**	0.476**	0.690**	0.627**	0.514**	0.564**	0.518**	0.630**	−0.048	0.660**	0.447**	–		1.95	0.433
STAI	0.494**	0.444**	0.506**	0.632**	0.570**	0.433**	0.587**	0.771**	0.631**	−0.049	0.711**	0.428**	0.659**	–	2.13	0.546

*N* = 612. *p < 0.05, **p < 0.01. ARCES, Attention-Related Cognitive Errors Scale; DFS, Daydreaming Frequency Scale; MAAS, Mindful Attention and Awareness Scale; CSS-IA, Current Symptoms Scale Inattentiveness Subscale; CSS-HI, Current Symptoms Scale Hyperactive–Impulsive Subscale; CTNES, Counterfactual Thinking for Negative Events Scale; PEPQ-R, Post-Event Processing Questionnaire-Revised; PSWQ, Penn State Worry Questionnaire; RRQ-Rum, Rumination-Reflection Questionnaire Rumination Subscale; RRQ-Refl, Rumination-Reflection Questionnaire Reflection Subscale; RRS-Brood, Rumination Responses Scale Brooding Subscale; RRS-Refl, Rumination Responses Scale Reflection Subscale; CES-D, Center for Epidemiologic Studies Depression Scale; STAI, State–Trait Anxiety Inventory; SD, Standard Deviation.

Next, a parallel analysis (PA) was conducted to assess a probable number of factors to extract. PA draws from a matrix of random values *via* data simulation from a population with the same number of variables and sample size as the current dataset, where variables are uncorrelated, compares current dataset scree plot to average scree plot from the simulated dataset, then estimates the number of factors by counting the number of eigenvalues that are greater than corresponding eigenvalues obtained from the simulated dataset (Zwick and Velicer, 1986; Hayton et al., 2004). Additionally, PA (Horn, 1965) has been documented to be most consistently accurate compared to previous common rules such as Kaiser’s ‘eigenvalue greater than 1’ (Kaiser, 1960) or Cattell’s (1966) scree plot (Franklin et al., 1995).

The PA generated from several different seeds for randomized iterations advocated for a 3–4 factor analyses for the current dataset. Both a three-and four-factor model EFA were conducted, using ordinary least squares extraction (due to non-normal distribution) and Oblimin rotation (due to theoretical expectation that our variables would be correlated). Although the four-factor model EFA demonstrated better fit indices (RMSEA of 0.068 [90% CI(0.054, 0.083)], TLI of 0.951, and *χ*^2^ (12, *N* = 612) = 92.33, *p* < 0.001), the three-factor model EFA that demonstrated fair fit indices (RMSEA of 0.098 [90% CI(0.086, 0.111)], TLI of 0.898, and *χ*^2^ (12, *N* = 612) = 228.34, *p* < 0.001) was selected for three main several reasons: (1) fit indices for EFA tends to be less reliable than in CFA/SEM contexts, (2) additional factors inevitably improves statistical fit to data, and (3) a more parsimonious model is desirable from a conceptual perspective (Finch, 2020). See Table 2 for factor loadings and percentage of total variance and see Table 3 for factor correlations.

**Table 2:** Factor loadings of inattention variables.

	Factor I	Factor II	Factor III
ARCES	**0.651**	0.143	0.054
MAAS	**0.520**	0.164	0.008
DFS	0.220	0.281	0.267
CSS-IA	**0.932**	−0.030	−0.030
CSS-HI	**0.833**	−0.042	0.042
RRQ-Rum	−0.071	**0.956**	0.050
PSWQ	0.060	**0.843**	−0.126
RRS-Brood	0.230	**0.466**	0.256
PEPQ-R	0.220	**0.445**	0.210
RRS-Refl	0.166	0.025	**0.714**
RRQ-Refl	−0.270	−0.040	**0.614**
CTNES	0.217	0.244	*0.369*
% of Total Variance	24.1	21.8	12.1

Loadings above 0.4 are bolded, loadings above 0.3 are italicized. ARCES, Attention-Related Cognitive Errors Scale; MAAS, Mindful Attention and Awareness Scale; DFS, Daydreaming Frequency Scale; CSS-IA, Current Symptoms Scale Inattentiveness Subscale; CSS-HI, Current Symptoms Scale Hyperactive–Impulsive Subscale; RRQ-Rum, Rumination-Reflection Questionnaire Rumination Subscale; PSWQ, Penn State Worry Questionnaire; RRS-Brood, Rumination Responses Scale Brooding Subscale; PEPQ-R, Post-Event Processing Questionnaire-Revised; RRS-Refl, Rumination Responses Scale Reflection Subscale; RRQ-Refl, Rumination-Reflection Questionnaire Reflection Subscale; CTNES, Counterfactual Thinking for Negative Events Scale.

**Table 3:** Factor correlations.

	Factor I	Factor II	Factor III
Factor I	–	–	–
Factor II	0.562	–	–
Factor III	0.377	0.440	–

### Objective 2

To determine whether recurrent thoughts mediate the relationship between MW and depressive and anxiety symptoms, structural equation modeling (also referred to as latent variable path analysis) was conducted. Although this model implies directionality, we cannot claim causation due to a cross-sectional study design. The main reasons for using SEM as opposed to regression are twofold. First, SEM allows a model to reflect underlying conceptual theory about latent constructs, and not only observed variables as in a regression. Secondly, SEM uses latent variables to account for measurement error, which also allows multiple indicators of the same construct, whereas multiple indicators in a regression model may cause collinearity problems (e.g., Gunzler et al., 2014). This SEM is not to validate factors as often done in confirmatory analysis, rather, to explore and establish covariation among the constructs of interest.

Findings from the above EFA were used to construct latent variables in the model to avoid issues of multicollinearity. Given that the measures that strongly loaded on Factor I (i.e., MAAS, ARCES, and CSS) assess the behavioral and cognitive consequences of inattention, we describe Factor 1 as “inattentive consequences” of MW. Although DFS is a commonly used MW measure, it weakly loaded on all factors and was therefore excluded. The *a priori* construct of recurrent thoughts loaded across two factors, separable into constructs of repetitive negative thoughts (RNTs; Factor II) and contemplative/reflective thoughts (Factor III). However, the latter construct was excluded altogether due to CTNES loading weakly on all factors and retaining only RRS-Refl and RRQ-Refl to form a latent variable on Factor III would result in model underidentification. Moreover, contemplative/reflective thinking is not central to the current research question and was therefore excluded. In turn, the remaining measures that strongly loaded on Factor II (i.e., RRQ-Rum, RRS-Brood, PSWQ, and PEPQ-R) are consistent with capturing RNTs (Ehring and Watkins, 2008; McEvoy et al., 2013; Zetsche et al., 2018), and thus we describe Factor II as RNTs. The two outcome variables were depressive symptoms (CES-D) and anxiety symptoms (STAI).

#### Finding 2

The mediation effect of RNTs on MW was explored using the lavaan package in R. Diagonally Weighted Least Squares (which implies Weighted Least Squares Means and Variances) estimator was selected due to non-normal data described under Finding 1. Standard errors of indirect effects were estimated using a bias-corrected bootstrapped standard error with 5,000 draws. In comparison, other methods of mediation tests (e.g., Baron and Kenny, 1986) have been shown to be statistically underpowered and less robust (MacKinnon et al., 2004). The current model fit was evaluated using several indicators. Although a chi-square test showed significant differences between the predicted model and the observed data, *χ*^2^ (31, *N* = 612) = 48.52, *p* = 0.023, this test is highly likely to be significant when sample sizes are large (*N* > 400) and when correlations are high, which conflicts with ideally conducting SEM on large sets of data. A combination of indices has been shown to result in the fewest Type I and Type II errors and thereby evaluating models as having reasonably good fit with consensus by various researchers (e.g., Browne and Cudeck, 1993; MacCallum et al., 1996; Steiger, 1998; Hu and Bentler, 1999; Schumacker and Lomax, 2010): (1) >0.96 TLI, (2) <0.06 RMSEA, and (3) <0.09 SRMR (Hu and Bentler, 1999). In the current dataset, TLI is 0.997, RMSEA is 0.030 90% CI (0.011, 0.046), *p* = 0.981, and SRMR is 0.041, all of which suggests that the model has a good fit overall.

**Figure 1:**
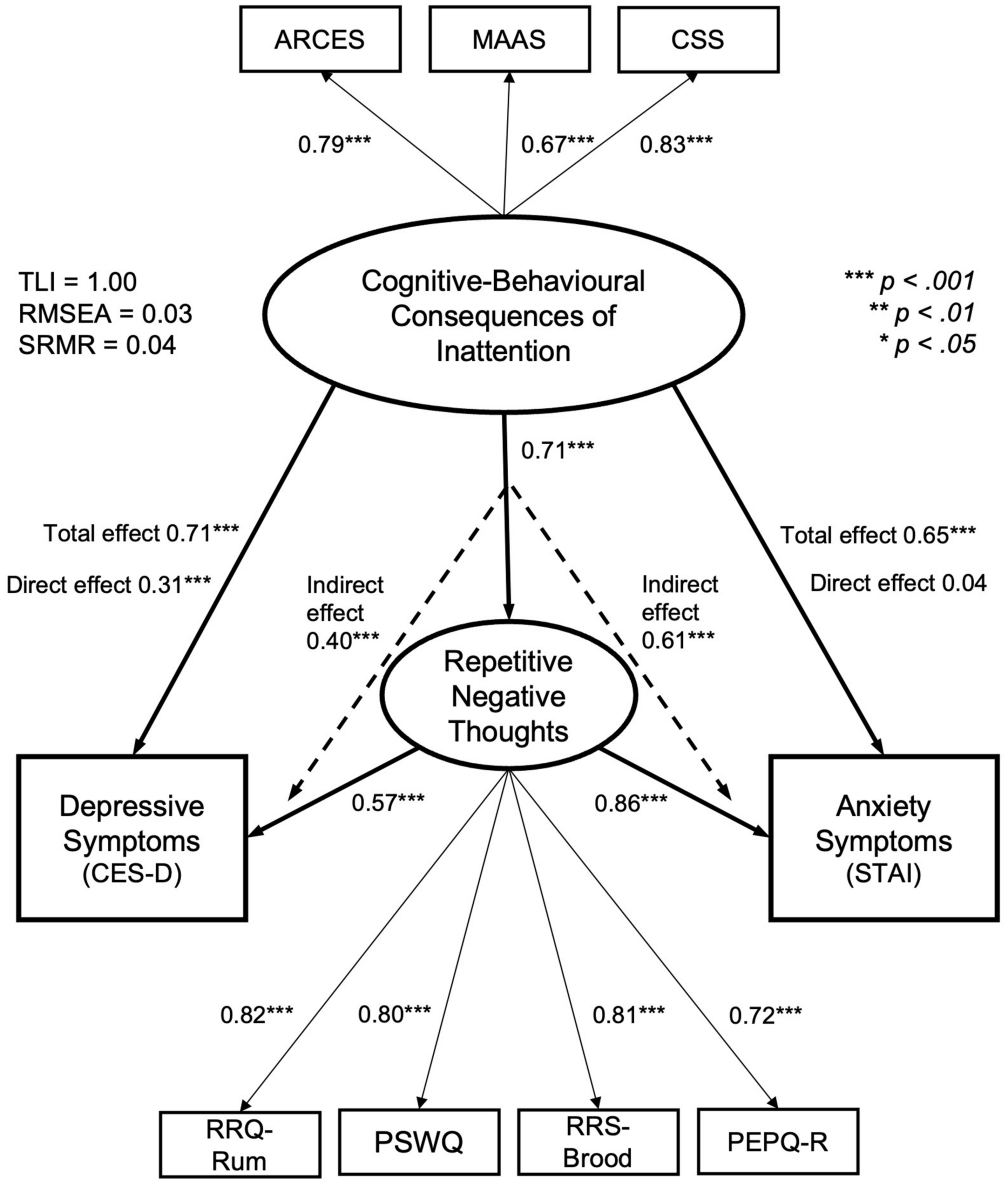
SEM model representing repetitive negative thoughts as a mediator in the relationship between cognitive-behavioral consequences of inattention and depressive/anxiety symptoms. *N* = 612. *p < 0.05, **p < 0.01, ***p < 0.001. Partial mediation of Repetitive Negative Thoughts (RNTs) was supported for consequences of inattention effect on depressive symptoms, and full mediation of RNTs was supported for consequences of inattention effect on anxiety symptoms. Standardized *ß*s are reported. ARCES, Attention-Related Cognitive Errors Scale; MAAS, Mindful Attention and Awareness Scale; CSS, Current Symptoms Scale; CES-D, Center for Epidemiologic Studies Depression Scale; STAI, State–Trait Anxiety Inventory; RRQ-Rum, Rumination-Reflection Questionnaire Rumination Subscale; PSWQ, Penn State Worry Questionnaire; RRS-Brood, Rumination Responses Scale Brooding Subscale; PEPQ-R, Post-Event Processing Questionnaire-Revised.

Given that CSS loaded strongly on Factor I with ARCES and MAAS in Finding 1, CSS was conceptualized post-hoc as part of the inattentive consequences latent variable. Indices TLI was 0.997, RMSEA was 0.030 with 90% CI (0.011, 0.046), and SRMR was 0.041, which indicated that the model has a good fit overall. Partial mediation was supported for the effect on depressive symptoms, as the significant total effect of MW/inattentive consequences on depressive symptoms (*b* = 0.462, 95% CI [0.418, 0.505]) can be separated into significant indirect effect (*b* = 0.262, 95% CI [0.211, 0.315]) and a significant direct effect (*ß* = 0.306, *b* = 0.200, 95% CI [0.134, 0.269]). Full mediation was supported for the effect on anxiety symptoms, as the significant total effect of MW on anxiety symptoms (*b* = 0.352, 95% CI [0.336, 0.411]) can be separated into a significant indirect effect (*b* = 0.332, 95% CI [0.313, 0.389]) and a non-significant direct effect (*ß* = 0.037, *b* = 0.020, 95% CI [−0.024, 0.063]). See Figure 1.

## Discussion

The main inquiry of the present study is to investigate the extent of consequences associated with various manifestations of inattention. Specifically, we examine how thought constructs converge with each other, particularly within MW and recurrent thoughts, as well as how this convergence may affect depression and anxiety symptoms. The first objective was focused on how MW and recurrent thoughts overlapped *via* commonly used self-report measures of each construct. The second objective examined how the interrelationship between MW and repetitive negative thinking (RNT) influences mood correlates.

The first set of findings demonstrated that these different manifestations of inattention fell across three factors. On Factor I, two measures of inattention (ARCES and MAAS) moderately to strongly clustered with each other; however, one measure of MW (DFS) only very weakly loaded onto this factor and a measure of ADHD (CSS) strongly clustered with the two MW measures. While the DFS is a more direct, face-valid measure of mind-wandering and did not load significantly on this factor, the three scales that loaded onto this factor are more accurately described as cognitive and behavioral correlates or consequences of mind-wandering or task-unrelated thoughts. Recalling that the ARCES was designed to be a measure of cognitive failures due to inattention, the MAAS, a measure of attention to and awareness of the present moment, and the CSS, a measure of symptoms manifested from ADHD, Factor 1 may be best interpreted as behavioral and cognitive consequences of inattention (henceforth, “inattentive consequences”). Although the MW field is progressing toward increased clarity in operationalizing MW and increased use of direct measures (Carriere et al., 2013; Mrazek et al., 2013; Kane et al., 2021), due to previous literature using a diverse set of indirect measures as proxies for MW (see reviews Schooler et al., 2011; Mooneyham and Schooler, 2013; Randall et al., 2014), our findings related to the inattentive consequences factor will be broadly discussed as MW below.

On Factor II, RNT expectedly converged with each other moderately to strongly. Measures that loaded strongly, RRQ-Rum and PSWQ, appear to inquire more generally about unwanted aspects and perceived uncontrollability of thoughts, compared to measures that loaded moderately, which appear to have a narrower scope of inquiry—RRQ-Brood focuses on content of the ruminative thoughts and PEPQ-R focuses on rehashing a specific social situation. On Factor III, reflection subscales of both RRS and RRQ load similarly alongside counterfactual thinking (CTNES), which loaded very weakly. These three measures appear to contain items that denote tendencies about introspection with some elements of deliberate intentionality for self-reflective thinking, further explored in sections below. Overall, this data-driven exploration of inattention suggest that MW/inattentive consequences, RNTs, and reflection/introspection may share common features but are also reasonably distinct constructs.

The second set of findings replicated results that showed MW/inattentive consequences and depressive/anxiety symptoms are related, and that inattentiveness and recurrent thoughts at least partially explained these relationships. SEM analysis based on the first set of findings to construct latent variables MW/inattentive consequences and RNTs demonstrated that the MW-depression relationship was *partially explained* by RNTs, and the MW-anxiety relationship was *fully explained* by RNTs. These results not only underlie the importance of considering other clinically-related thoughts when studying MW and affect/mood, but also the importance of considering other MW operationalizations that will vary depending on the specific research inquiry (for differing perspectives on how MW may be construed, see Christoff et al., 2016; Seli et al., 2018).

These findings above may also broadly inform the dimensional nature of inattention. Three important considerations are discussed below: intentionality of inattention, executive control models of inattention, and the thought content of inattention.

### Intentionality of inattention

Although MW research appear to assume that we have limited control over thoughts that enter and remain in our minds, evident in self-report measure items, distinguishing deliberate versus spontaneous MW has been useful in showing some potential differences in mood correlates.

On the one hand, as mentioned previously, depression, anxiety, and stress appear to be more common in spontaneous MW compared to deliberate MW (Seli et al., 2019); however, evidence remains mixed in that individuals who have more severe depressive symptoms mind-wander more frequently both with and without meta-awareness (Nayda and Takarangi, 2021), implying deliberate and spontaneous MW, respectively. Spontaneous MW is also associated more strongly with ADHD symptomatology and associated impairments more so than deliberate MW (Seli et al., 2015). In the current study, both MW/inattentiveness (Factor I) and RNT (Factor II) comprises measures with items that suggest unintentional or spontaneous thinking, whereas reflection/introspection (Factor III) comprises measures with items that suggest intentional or deliberate analytical thinking. Moreover, the measures that constitute Factor III appear to have a weaker correlation with negative mood symptoms, largely driven by RRQ-Refl, which appears to contain items with higher face validity in inquiring about reflection and contemplation independently from low mood compared to RRS-Refl. Futuremeasures or studies of inattention may be more cognizant of such an underlying assumption of awareness or intentionality, and use scales such as the brief Mind Wandering: Deliberate scale and Mind Wandering: Spontaneous scale, which clearly demarcates differences between intentionality of MW *via* self-report (e.g., “I allow my thoughts to wander on purpose” vs. “I find my thoughts wandering spontaneously”; Carriere et al., 2013), or the Rumination scale, which demarcates intrusive and deliberate/constructive rumination (e.g., “I thought about the event when I did not mean to” vs. “I have tried to make something good come out of my struggle”; Calhoun et al., 2000).

On the other hand, rumination and MW research findings appear to be more consistent, suggesting that intentional thoughts may not be systematically associated with negative outcomes unlike unintentional thoughts. Ruminative thoughts have been characterized as “brooding” rumination and “reflective pondering” (Treynor et al., 2003) as well as “intrusive” and “deliberate” rumination (e.g., Taku et al., 2009). Reflective pondering and deliberate rumination tend to be purposeful, constructive problem-solving, qualities of intentional thinking, whereas brooding and intrusive rumination tend to be passive, non-constructive dwelling, qualities of unintentional thinking. Considered together, intentional rumination (reflective and deliberate) appears to have a stronger positive correlation with post-traumatic growth (Taku et al., 2009; Stockton et al., 2011), in contrast, unintentional rumination (brooding and intrusive) appears to have a stronger positive correlation with negative affect (Moberly and Watkins, 2008), depression (Burwell and Shirk, 2007), and suicidal ideation (Chan et al., 2009). Moreover, a small body of research distinguishes between “cued” and spontaneous counterfactual thoughts, noting that more depressed individuals may have lowered ability to spontaneously develop alternative courses of action (*via* counterfactual thinking) compared to their less depressed counterparts (Beldarrain et al., 2005). Although many of the recurrent thoughts included in this present study have not explicitly distinguished between intentional and unintentional aspects of the constructs, the second set of findings suggest that consequential differences between MW and recurrent thoughts are less likely to be due to intentionality dimensions, which may find its mechanisms explained in executive functioning.

### Executive functioning models of inattention

The finding that MW/inattentive consequences and RNTs fell into different factors suggest there may be meaningful differences between the two broader constructs, a similar inference with the intentionality domain above. Further, the finding that RNTs at least partially mediate the relationship between MW and both depression and anxiety suggests that executive mechanisms implicated in MW could overlap with executive mechanisms implicated in RNTs. But if so, to what extent might this possibility align with predominant theories linking executive function to MW, inattention, and RNTs?

Executive functioning models in MW and ADHD do have some convergence, even though they do not thoroughly overlap partially due to MW being a relatively narrow cognitive phenomenon, whereas ADHD involves a large set of symptoms with inattentiveness and hyperactivity/impulsiveness subtypes. Notably, despite a handful of studies showing associations between MW and one or more domains of ADHD (i.e., inattention, hyperactivity, impulsivity; e.g., Biederman et al., 2017; Jonkman et al., 2017; Arabaci and Parris, 2018), none of these relationships have been specifically linked with executive functioning and/or mechanistic explanations of these processes (Lanier et al., 2021). Nonetheless, the nature of inattention can be conceptualized as a broad spectrum. In particular, MW has been associated with poorer performance on a concurrent task and has been explained by either MW consuming working memory resources (Smallwood and Schooler, 2006), or MW representing a brief lapse in executive control (McVay and Kane, 2010). ADHD executive functioning deficits have been explained by the Inhibition model, which proposes that a primary impairment of inhibition sets the stage for secondary executive functioning deficits to surface, such as working memory (Barkley, 1997). ADHD executive functioning deficits have also been explained by the Cognitive-Energetic Model, which proposes an interplay among three levels of difficulties: (1) a lower-level attentional control such as encoding, (2) a second-level energetic system related to arousal, and (3) a higher-level executive functioning system. This highest level has been described as a system that maintains “problem sets” for goal-directed behaviors (Sergeant, 2000).

Although these two models of MW and two models of ADHD are all distinct, they are nonetheless congruent in several critical ways. For one, the working memory consumption model of MW aligns with the Inhibition model directly *via* the proposed secondary deficiency of working memory, and likewise, the executive functioning lapse model of MW aligns *via* the primary deficiency of inhibition that leads to an overall executive functioning lapse. Moreover, both MW models align with and are relevant to the Cognitive-Energetic Model, which proposes executive management failures are related to a low ability to keep goals in mind, and therefore is relevant for working memory consumption as well as executive control more broadly.

Similar convergence is seen when considering theorizing on RNTs. To the point, there are four main RNT models related to executive functioning that can complement or extend MW models. Three of these models suggest that impaired executive functioning underlies RNTs and prevents regulation of thoughts, and in particular, through either lowered ability to (1) update working memory to include positive or goal-directed information (Whitmer and Gotlib, 2013), (2) inhibit negative or goal-irrelevant information from returning into working memory (Joormann and Gotlib, 2010), or to (3) shift attention or flexibly disengage from negative, goal-irrelevant information (Koster et al., 2011). The fourth model linking RNTs to executive function, however, suggests the reverse direction, and specifically, that RNTs deplete cognitive resources for optimal executive functioning, leading in turn to lowered ability to shift attention away from perceived threat and lowered inhibition of irrelevant information (Eysenck et al., 2007; Stefanopoulou et al., 2014). To be certain, while the first three models focused on rumination and the fourth model focused on worry, they may all apply to RNTs in general due to RNTs’ transdiagnostic nature (Ehring and Watkins, 2008; Wahl et al., 2019), frequent comorbidities for mood and anxiety disorders (McEvoy et al., 2013), and common co-conceptualization (see review Mennies et al., 2021).

Converging evidence comes from counterfactual thinking. Classified in this study as a type of recurrent thought, though ultimately excluded from the latent construct in analyses, counterfactual thinking also involves holding a different timeline than the present reality in working memory, comparing alternative courses of actions and outcomes, and actively monitoring various executive functions (e.g., Kahneman and Miller, 1986; Roese, 1997; Ridderinkhof et al., 2004; Van Overwalle, 2009; Barbey and Patterson, 2011; Van Hoeck et al., 2013). Along the same lines, adaptive qualities are evident in rumination, persistently seeking resolutions to personal concerns (Klinger, 1971, 2012), and in worry, alerting attention toward perceived threat (Hirsch and Mathews, 2012). The proposal that recurrent thoughts occur and are maintained to keep personally relevant issues in mind may be directly applicable in consuming excessive working memory in the working memory depletion model of MW. Furthermore, the maintenance of recurrent thoughts may be integrated into the executive functioning lapse model of MW, such that the attentional lapse is explained by goals of competing importance at any given moment—the current task at hand vs. personal concerns or perceived threats—rather than an executive “conflict,” rather than “failure,” *per se*.

Overall, executive functioning may be the essential mechanism that assists in switching from abstract thinking in recurrent thoughts to concrete problem-solving, accurately appraising potential threats, and increasing meta-awareness in recognizing and intentionally deciding upon current goals when our thoughts have drifted. Also given the finding that executive functioning deficits have been shown to moderate the association between RNTs and negative affect, i.e., those with weaker executive functioning had more RNTs and higher negative affect, but those with stronger executive functioning had lower negative affect even with presence of RNTs (Madian et al., 2019), future research may need to consider examining individual differences in higher vs. lower executive functioning related to MW or RNTs and negative mood to further illuminate executive functioning consequences of inattention.

### Thought content of inattention

The content of thoughts is often one of the most salient features when describing different types of inattentive thoughts; however, this qualitative nature of thoughts is not consistently assessed across measures. As we consider the relationship between mood and MW, naturally, we would expect the subject matter of our thoughts to influence how we feel. Many studies in earlier research have explored MW content using experience sampling or thought probes, in which participants are interrupted throughout a given period. At each interruption, they would report what they had been just thinking about—ranging from open-ended responses to assigning thoughts into at one or more categories of future/past, self/other, positive/negative (e.g., Killingsworth and Gilbert, 2010; Smallwood and O'Connor, 2011; Shrimpton et al., 2017). Although these studies that probe the content of in-the-moment MW thoughts are informative in the “what” of inattention, it appears that many MW-related questionnaires about thoughts lean more toward querying the “how” of inattention; that is, the quality and characteristics of these thoughts rather than specific content *per se*, which may be cognitively informative at a deeper mechanistic level.

The “how” of inattention—how thoughts characteristically unfold or progress overtime—is informative for understanding thoughts processes, such as how thoughts become fleeting, maintained, or intensified, or how certain thinking styles or “habits” may be formed (or learnt) based on how frequently these mechanisms are repeated. In contrast, the “what” of inattention—what the specific substance of the thoughts are about at any given moment—provides a cross-sectional observation of only the output emerged or materialized from various dynamic thought processes that are based on countless possible inputs and pathways. In other words, whether thoughts are persistent and repetitive or meandering and free flowing, or whether they are generalized across situations or limited to specific situations, may be more illuminating than the object or subject of inattentionbecause examining thought patterns reveal the means rather than the end outcome (i.e., thought content), and may unveil more about any given individuals’ mind.

Using questionnaires in the present study as exemplars, rather than inquiring about subject matter during inattention, the “how” of mind wandering/inattention is assessed by behavioral outcomes or impacts of inattention such as forgetting information or tasks, day-to-day recurring mistakes. Comparably, the “how” of RNT subscales or measures is assessed by thinking styles or patterns such as the frequency, unpleasantness, and perceived uncontrollability of such cyclical thoughts. The “how” of reflection/contemplation subscales is assessed by the level of enjoyment one derives from analytically introspecting. Overall, while self-report surveys may be simplified as generally assessing trait-based qualities, and self-report lab probes assessing state-based real-time thoughts, beyond a multimethod approach *per se*, researchers may appreciate the “how” data gathered that can generate and inform research questions about inattention and associated consequences in general.

### Limitations and future directions

From a statistical point of view, a broad limitation is that factor analyses and related model development require a considerable amount of subjectivity to determine fit into either novel or existing theory. Specific to SEM, researchers have been cautioned against using cross-sectional mediation analyses due to biased estimates produced (e.g., Maxwell and Cole, 2007; Maxwell et al., 2011). The current study aims to preliminarily test and demonstrate that the construct of MW may heavily overlap with construct(s) of recurrent thought, and this has been replicated yet again correlationally. Nonetheless, although our model fit the data well based on the preceding EFA conducted, the generalizability of our data will need to be tested through replication. In addition, the cross-sectional nature of this investigation is a limitation for future research to examine causality, that is, whether propensity to mind-wander precedes susceptibility to recurrent thoughts, and consequently leads to negative mood symptoms. A data-driven approach may be useful in exploring underlying constructs or characteristics of inattention. However, the findings and conclusions reported here are undoubtedly limited by measures included in the study, which was not comprehensive or an exhaustive list. For example, regarding MW measures, three commonly used scales were selected to understand how MW has been frequently operationalized, but future studies may consider including questionnaires that have been more recently developed, such as the Mind-Wandering Scale: Deliberate and the Mind-Wandering Scale: Spontaneous (Carriere et al., 2013). Such next-generation scales suggest further consideration of different construals of MW may be fruitful, in terms of how they may or may not be complementary. Regarding RNTs and other clinically based measures, future studies may also want to consider “intrusive thoughts,” typically associated with obsessive thoughts. Likewise, when studying MW and its relationship to negative mood, it may also be best practice to include internal thoughts that are strongly correlated with low mood such as RNTs included in the present study.

Moreover, the many recurrent thoughts included in this study are associated with negative affect, and future studies may consider adding to the affective range when exploring inattention, such as thought constructs that are more neutral or positive (e.g., fantasy proneness; Merckelbach et al., 2001). In extension to affect, another next step may be to investigate how various types of off-task thinking operate in relation to each other, or in the presence of each other; for example, while inattention in the form of recurrent thoughts, especially RNTs, may be more related to affect consequences, inattention in the form of planning may be more related to cognitive consequences. Lastly, a study involving self-report of cognition will fall prey to issues of limited meta-awareness; nonetheless, as researchers delve further into inattention and its correlates, the relation between MW and negative moods can be clarified, and the mind’s narrative thinking processes may be ultimately uncovered to reveal unhelpful patterns of thinking and ameliorate the multidimensional consequences of inattention.

## Data availability statement

The raw data supporting the conclusions of this article will be made available by the authors, without undue reservation.

## Ethics statement

The studies involving human participants were reviewed and approved by University of British Columbia Behavioural Research Ethics Board. The patients/participants provided their written informed consent to participate in this study.

## Author contributions

JY and TH contributed to the conception, design of the study, and interpreted data. JY acquired the data, analyzed the data, and drafted several drafts. NJ assisted with organizing and cleaning the dataset, literature reviews, and gathering references. TH critically reviewed and revised the manuscript and provided substantial intellectual content. All authors contributed to manuscript revision, read, and approved the submitted version.

## Funding

This study was funded by Natural Sciences and Engineering Research Council of Canada, award F19-04992.

## Conflict of interest

The authors declare that the research was conducted in the absence of any commercial or financial relationships that could be construed as a potential conflict of interest.

## Publisher’s note

All claims expressed in this article are solely those of the authors and do not necessarily represent those of their affiliated organizations, or those of the publisher, the editors and the reviewers. Any product that may be evaluated in this article, or claim that may be made by its manufacturer, is not guaranteed or endorsed by the publisher.
